# Stroke 1-2-0: The strategy and video release

**Published:** 2017-02-14

**Authors:** Jing Zhao, Renyu Liu

**Affiliations:** 1 Department of Neurology, Minhang Hospital, Fudan University, Shanghai, China; 2 Department of Anesthesiology and Critical Care, Perelman School of Medicine at the University of Pennsylvania, Philadelphia, PA USA

To reduce prehospital delay for stroke victims in China is very critical and urgent as it is one of the major factors for the highest mortality and disability rate in China.^1-3^ Our proposal to use Stroke 1-2-0 strategy for rapid stroke recognition and response is very timely. Immediately after the online publication of *Stroke 1-2-0* in Lancet Neurology^4^, the Chinese Stroke Association (CSA) endorsed it on World Stroke Day, Oct 29, 2016. Massive media coverage by over 50 regional and national news agencies followed. China Central Television broadcasted a special introduction of *Stroke 1-2-0* nationwide. After a few hours of its broadcast, the social media account (weibo) for Chinese Central Television had over 101 million people reviewed the introduction of Stroke 1-2-0.

To further promote *Stroke 1-2-0* in China, we are currently working closely with CSA to develop an effective plan. CSA is establishing a Stroke 1-2-0 special task force of Chinese Stroke Association lead by both Dr. Jing Zhao and Dr. Renyu Liu. This is the first time for CSA to establish such special task since the prehospital stay is considered as the major factor in driving the high mortality and disability rate for stroke victims in China. We believe that such special task force will reduce prehospital delay significantly by promoting Stroke 1-2-0, improving awareness of stroke in the public domain with immediate action by calling medical emergency service1-2-0. Figure 1 shows the special log for this new special task force. In this logo with highlighted Stroke 1-2-0, there is a ticking clock to the right to indicate the urgency of the situation. The brain within the clock indicating more brain loss will occur when the time is ticking. The reverse arrow indicates our ambition to reduce the pre-hospital delay and the brain injury when stroke occurs.

Stroke 1-2-0 is now incorporated into the practice guideline for thrombolytic therapy in China by CSA to be released in 2017. A dedicated website (www.stroke120.org) and a social media Wechat public platform (Chinastroke120, www.wechat.com) were established to deliver the most updated information related to *Stroke 1-2-0*. A *Stroke 1-2-0* educational video is produced by us and is released to the public domain via our website (http://stroke120.org/animation/) and many other media platforms. After the successful introduction, we strongly feel that it is critical to create a video for an easy understanding of the Stroke 1-2-0 program. In this short 1 min video (Video 1.), we deliver the message that stroke is an acute and severe disease that can cause life-long disability and even death if the disease is not treated in a timely manner. To have the disease to be treated in a timely manner, it is critical to recognize the stroke and trigger the medical emergency system immediately. How to recognize the stroke signs and symptoms are well presented using Stroke 1-2-0 strategy with very simple and easy understanding animations. Dr. Jing Zhao acted as a physician in this video, she also presented the voice to teach how to use Stroke 1-2-0 for recognition of stroke.

It is critical to note that when one has a cardiac infarction (a heart attack), another type of medical emergency, patient generally has very severe angina (chest pain) and pending doom, the patient is more likely to call or to ask people around the victims to call medical emergency service. However, when one has an ischemic stroke, it is more silent, patient may not be aware of the severity of the problem, and unlikely to trigger a medical emergency call. There is no rescue medication that a patient or patient's family member(s) can carry with to deal with such emergency situations. Thus, to trigger medical emergency system is very critical. Another important fact is that once the medical emergency system is alerted, a nearby stroke care system or team can be alerted also via the medical emergency system. Thus, the patient can go through the special stroke care path (green path for stroke victim) to avoid delays in hospital.

## Supplementary Material

video 1 The Stroke 1-2-0 educational video endorsed by the Chinese Stroke Association and the special task force.

## Figures and Tables

**Fig. 1:**
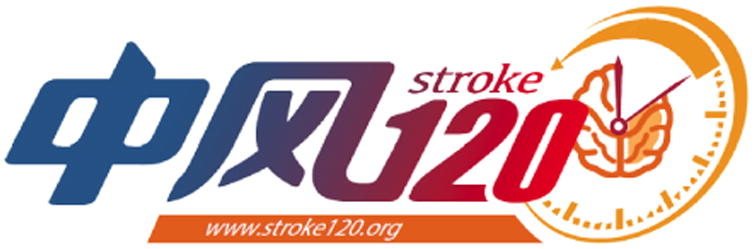
The logo for the Special Task Force of the Chinese Stroke Association.
